# The relational ground of emotion: an Ubuntu-based critique of individualist ontology in theories of emotion

**DOI:** 10.3389/fpsyg.2026.1926813

**Published:** 2026-09-14

**Authors:** Şeyma Sevgican Yardımcı

**Affiliations:** Department of Psychology, Hasan Kalyoncu University, Gaziantep, Türkiye

**Keywords:** collective emotion, cultural psychology, decolonial psychology, emotion, ritual-based emotion, Ubuntu philosophy

## Abstract

Emotion studies increasingly recognize the decisive role of cultural context in emotional experience. However, this acceptance rests on an unquestioned ontological assumption that an individual subject experiences emotions, and that culture shapes this subject from the outside. This study aims to ontologically challenge this assumption within the context of small-scale traditional communities in Africa. This study, conducted using critical review methodology and PRISMA 2020 reporting standards, searched the Web of Science, Scopus, and PsycINFO databases. 57 qualitative and theoretical studies meeting the inclusion criteria were evaluated. The included studies address ritual practices, collective identity, social norms, and cosmological belief systems as cultural contexts of emotion. The findings reveal that emotions in these communities are not individual experiences shaped by culture, but relational processes rooted in collective networks. The Ubuntu philosophy principle, “A person is a person through other persons,” ontologically positions emotional experience as inseparable from collective existence. These findings indicate that the individualistic ontology underlying current emotion theories is not only a cultural limitation but also an epistemological constraint of the field. An Ubuntu-centered theoretical model comprising four components (relational ontology, ritualistic-performative emotion production, cosmological appraisal, and decolonial epistemology) is proposed alongside eight testable propositions for future empirical research.

## 1 Introduction

Over the past 30 years, the psychology of emotion has increasingly acknowledged the determinative role of cultural context. However, this acknowledgment itself rests on a specific ontological assumption: that there is an individual experiencing emotion and that culture shapes this individual from the outside. From Markus and Kitayama (1991) to Barrett (2017), culture has been positioned as the context, content, or means of emotion; however, the individual nature of the subject experiencing emotion has not been questioned. This study aims to re-evaluate the theoretical framework of the psychology of emotion, starting from a context that ontologically questions this assumption: small-scale traditional African communities. However, the proposal of this study is not merely a change in cultural context. Concepts such as collective emotion, relational self, or cultural norm already exist in the current literature. Therefore, the claim of this study points more to structural problems. None of these concepts question the individual nature of the subject experiencing emotion. While even theories of collective emotion ultimately attribute the emotional process to individuals, Ubuntu philosophy removes this foundation at the ontological level. This difference explains why existing theoretical frameworks fail to fully grasp the African context.

According to Henrich et al. (2010), 96% of the samples in behavioral science literature come from WEIRD (Western, educated, industrialized, wealthy, and democratic) countries. However, this population represents only 12% of the world’s total population. Mesquita (2022) introduced a new perspective to the field by arguing that emotions arise not from within the individual, but from relationships. However, the existing literature is largely confined to the East–West dichotomy, and ritual-based, small-scale communities remain outside the scope of systematic psychological analysis. Small-scale traditional communities in Africa offer a particularly unique context to fill this gap. In these communities, emotions appear not as the individual’s inner experience, but as complex systems of meaning shaped by social norms, rituals, collective identity structures, and the Ubuntu philosophy (Eze, 2014; Oppong, 2022). For example, the song-like norms of the Akan people and concepts like safuu of the Borana Oromo, just like the local emotion categories documented by Lutz (1988) in the Ifaluk society, directly challenge the universality claim of Basic Emotions Theory and the individualistic assumptions of Cognitive Appraisal Theories (Crivelli et al., 2016; Gendron et al., 2012).

Before moving on to the main objective of this study, it is necessary to clarify the terminology. The concept of “tribe” is criticized in contemporary social anthropology and decolonial studies literature as a hierarchical label produced by the colonial perspective (Mudimbe, 1988). In this study, the communities mentioned are defined as “small-scale traditional communities” or “indigenous communities,” and the term “tribe” is used only in contexts that adhere to the original terminology of the source studies. In addition, the term “ontology” does not imply that emotions exist as fixed essences. On the contrary, the understanding adopted here is closer to the tradition of process ontology (Whitehead, 1978), which emphasizes that being is composed not of static substances, but of relationships in a constant state of becoming. Therefore, the phrase “ontological ground of emotion” is not a fixed definition of what emotions are, but a positioning of the level at which emotional experience arises. Indeed, this distinction is critical. While constructivist approaches argue that emotions are constructed, they position the subject of the construction process within the individual brain and body. The Ubuntu perspective does not reject this construction process; it offers a different answer regarding where the construction takes place. Emotion is still constructed, but the constructing unit is not the individual organism, but the relational network itself. In other words, this study’s objection is not to constructionism itself, but to its methodological individualism.

This study aims to simultaneously close three theoretical gaps: (1) addressing the WEIRD bias in the psychology of emotion not only at the sample level but also at the ontological level; (2) systematically relating the literature on collective emotion (Barsade, 2002; Hatfield et al., 1993; Von Scheve and Salmela, 2014) to the African context; (3) Showing why the integration of Barrett’s (2017) constructivist framework and Boiger and Mesquita’s (2012) cultural norm model with the Ubuntu ontology is partly possible but structurally inadequate. This study focuses on four theoretical frameworks: Ekman’s (1992) Basic Emotion Theory; Lazarus and Smith’s (1988) and Ellsworth and Scherer’s (2003) Cognitive Appraisal Theories; Barrett’s (2017) Constructivist Emotion Theory; and Boiger and Mesquita’s (2012) Cultural Norm Theory. These four theories represent the mainstream theoretical spectrum of emotion psychology, carrying differing assumptions regarding fundamental tensions between universality and cultural specificity, biology and construction, and the individual and relational processes. This theoretical diversity provides a necessary theoretical foundation for a comparative analysis of the extent and mechanisms by which the African context challenges individualistic ontology. In selecting the theories, three criteria were considered: paradigmatic weight, measured by citation impact and coverage in textbooks; diversity among ontological positions; and actual application in cross-cultural emotion research. Dimensional models, psychoevolutionary approaches, and phenomenological perspectives were excluded, and these restrictions are discussed in the limitations section. Below, these four theories are discussed in turn; for each, their explanatory capacity and limitations within the African context are evaluated.

### 1.1 Research questions

This study seeks to answer three fundamental, complementary questions:

RQ1: In what ontological framework are emotions conceptualized in small-scale traditional African communities, and with what socio-cultural meanings are they associated?

RQ2: To what extent are Basic Emotion Theory, Cognitive Evaluation Theories, Constructivist Emotion Theory, and Cultural Norm Theory sufficient or insufficient in explaining emotional patterns in these communities?

RQ3: Based on these findings, what theoretical components are necessary to develop a theory of emotion that goes beyond individualistic ontology and has cross-cultural validity; what conceptual and methodological limitations do Western-centric measurement tools have in these contexts?

## 2 Conceptual framework

### 2.1 Historical and theoretical basis of emotion

Emotions have been central to efforts to understand human nature from Ancient Greece to the present day. The process, which began with Aristotle’s concept of pathe, continued with Plato’s placement of emotion in opposition to reason in his mind–body dualism, and, with the birth of experimental psychology in the late 19th century, became the primary object of scientific analysis (Power and Dalgleish, 2008). Darwin's (1897) evolutionary-based work and James's (1884) article “What Is an Emotion?” permanently connected emotion with the science of psychology. Today, theories of emotion are organized around two main axes. The first axis addresses whether emotions are categorical or dimensional. Ekman's (1992) categorical approach argues that universal biological circuits produce a limited number of basic emotions; Russell's (1980) Circumplex Model positions emotions along valence (positive–negative) and arousal (high-low) dimensions. Barrett's (2017) concept of core affect combines this dimensional tradition with constructivism, suggesting that bodily sensations are transformed into categories through cultural concepts. For the African context, which is the focus of this study, the dimensional approach is considered particularly critical. If concepts like *nagaa* or *isinyama* cannot be reduced to valence-arousal dimensions, this represents not merely a cultural variation, but an ontological objection to the claim of dimensional universality.

The second axis addresses whether emotion is individual or relational. Cowen and Keltner (2017), in their study with thousands of participants, identified 27 distinct emotion categories, demonstrating the inadequacy of both Ekman’s six-dimensional classification and Russell’s two-dimensional model. Mesquita (2022), on the other hand, argues that emotions arise not within the individual but within relationships, proposing a fundamental break from this second axis. Small-scale African communities achieve this break not at the level of individual preference or cultural difference, but at the ontological level.

The field of collective emotion research, which has been gaining strength over the last decade, demonstrates that emotions are not individual experiences but group-level constructs. The collective emotion literature compiled by Von Scheve and Salmela (2014) spans a wide theoretical spectrum, from emotional contagion (Hatfield et al., 1993) to collective enthusiasm (Durkheim, 1995), from ritual synchronization (Whitehouse, 2004) to group-level emotion regulation. However, small-scale African communities carry out this collective process not at the level of individual preference or cultural variation, but by positioning the relational network itself as the ontological ground of emotion. This difference is crucial to understanding why existing theories of collective emotion only partially explain, and do not fully grasp the African context.

### 2.2 Basic emotions theory

The Basic Emotions Theory (Ekman, 1992) argues that six basic emotions (happiness, anger, fear, sadness, disgust, and surprise) are present in all humans as a biological endowment and are expressed through cross-culturally consistent facial expressions. The theory’s fundamental assumption is biological essentialism.

In this study, this assumption is questioned at two levels: empirical and ontological. At the empirical level, neuroscientific data from Jack et al. (2012) revealed that the six basic emotion categories of the West were perceived as only four categories by East Asian participants. Study findings by Crivelli et al. (2016) showed that a facial expression coded as “fear” was interpreted as “threat” or “aggression” by Trobriand Islanders. Gendron et al. (2012) empirically demonstrated that even universally accepted facial expressions do not carry a common meaning without linguistic categories. Despite the potential expansions of the theory’s most sophisticated proponents, including Tracy and Robins' (2004) work on pride and Cowen and Keltner's (2017) proposal of 27 categories of emotion, the following structural problem remains unanswered: “Regardless of the extent to which these expansions are realized, the individual biological nature of the subject experiencing emotion remains presupposed.” Categories such as *nagaa* or *collective enthusiasm* can only be explained by re-establishing the ontological ground, not by expanding the biological basis.

That said, it is important to emphasize that this critique does not negate the role of physiological and biological processes in emotional experience. Bodily arousal, autonomic responses, and neural processing are undeniably involved in the emergence of emotions. The objection in this research is directed at the assumption that these processes necessitate the individual organism as the ontological unit of emotion. Physiological processes can and do operate within relational networks. The question is not whether biology matters, but whether conceptualizing bodily existence within individual boundaries is necessary.

### 2.3 Cognitive appraisal theories

Cognitive appraisal theories (Ellsworth and Scherer, 2003; Lazarus and Smith, 1988) suggest that emotions stem from subjective evaluations of the meaning of environmental stimuli for the individual. However, classical models address the evaluating subject through a Western-centric, autonomous, and individualistic self. However, Markus and Kitayama's (1991) conceptualization of the “interdependent self” functions as a critical analytical tool in this debate. In the African context, appraisal is made not only according to collective goals but also according to the ancestor-human relationship, cosmological balance, and spiritual obligations.

This study argues that this expansion must necessarily include the axes of “collective cognitive appraisal,” “ancestor-human relationship,” and “cosmological balance.” However, to strengthen this defense theoretically, it is necessary to consider the most advanced forms of Cognitive Appraisal Theories [e.g., Scherer's (2001) Component Process Model]. Scherer’s model opens the door to partially accommodating cosmological evaluation through the “normative conformity” appraisal dimension. However, this door is locked by the assumption of an individual-subject evaluation. Scherer’s model also falls short when the subject of appraisal is the relational network itself, as in the Ubuntu ontology.

### 2.4 Constructivist theory of emotion

Barrett (2017) argues that emotions are constructed through the instantaneous interpretation of bodily sensations, mediated by cultural concepts and language. Lindquist's (2013) constructivist model provides a neuroscientific basis for the approach by showing that the use of the concept of emotion varies across cultures and that this variation can be traced in brain imaging. The African context also offers the strongest empirical support for this theory. Turner (2020) emphasized that in Algerian dīwān rituals, collective suffering is transformed through music and bodily practices. Similarly, Yepthomi et al. (2025) showed that emotional states are constructed through local narratives in Naga tribes. However, the criticism that Barrett ignores the predictive processing framework’s potential is one of this study’s main arguments. In short, even the predictive processing framework continues to center an individual brain as the subject of construction. In the Ubuntu ontology, the subject of construction is the relational network itself. This difference can be overcome not by expanding the scope of the predictive processing model, but by ontologically repositioning it. Translating Barrett’s concept of “core affect” into relational networks is theoretically not impossible and provides a fruitful dialogue agenda for future studies, but this step has not yet been taken.

### 2.5 Cultural norm theory

Boiger and Mesquita (2012) argue that emotions are regulated and gain meaning within social relationships and cultural norms rather than as an individual inner experience. Vishkin and Tamir (2023) have shown that norms target not only the display of emotion but also the subjective experience itself. Matsumoto et al.'s (2008) study across 31 countries revealed that in collectivist cultures, the suppression of negative emotions is a normative necessity for maintaining in-group harmony. Joshanloo and Weijers (2025) have shown that even positive emotions, such as happiness, function as social signals demonstrating loyalty to the group. This theory best aligns with the African context among the four theories, as it positions emotion as a relational process from the outset. However, it fails to critically grasp situations where norms are instrumentalized for patriarchal structures, bodily-harming rituals, or structural violence. More fundamentally, this theory does not question the individual nature of the subject experiencing emotion. In Mesquita’s model, emotion arises from the interaction between individuals; that is, the relationship is established between two pre-existing subjects. In the Ubuntu perspective, however, the relational network ontologically precedes; individual subjectivity is a derivative of this network. This is the difference between *“emotion is relational”* and *“relationship is the ground of emotion and subjectivity.”*

Another distinction is the exclusion of the cosmological dimension. Mesquita’s model proceeds through social interaction, cultural norms, and linguistic differences. Evaluative dimensions such as ancestor-human relations, cosmic balance, and spiritual obligation remain outside the scope of this framework. Yet, in the communities studied, these dimensions are central components of emotional experience. Finally, the sociodynamic model, while describing cultural differences, does not question within which epistemic framework these differences are defined. The structural impact of the colonial legacy on both patterns of emotion and the conceptual framework of emotion research remains invisible without a decolonial framework (Mudimbe, 1988; Oppong, 2022). In short, Mesquita’s contribution is to move emotion from the individual to the relationship; this work proposes to position the relationship itself as the ontological ground and to expand this ground with cosmological and epistemological dimensions.

### 2.6 From individual skill to collective achievement: ontological assumptions of emotion regulation

Within the science of emotions, emotion regulation has become a research field in its own right over the last two decades. While the four theories examined above focus on the questions of what emotion is and how it arises, the emotion regulation literature addresses how emotion is modified, managed, and transformed. Including this field within the theoretical framework broadens the scope of the critique of individualistic ontology.

In the emotion regulation literature, Gross's (2015) extended process model forms the dominant framework of the field. The model addresses emotion regulation in five stages: situation selection, situation modification, attentional deployment, cognitive change, and response modulation. Each stage involves an evaluation cycle that determines the regulation goal, selects the appropriate strategy, and implements it. Cross-cultural studies show that the frequency and effectiveness of these strategies differ according to cultural context. For example, cognitive re-evaluation stands out as an adaptive strategy in Western samples, while the negative effects of the suppression strategy are significantly reduced in collectivist contexts (Matsumoto et al., 2008). These findings acknowledge cultural variability but do not question the fundamental structure of the model. Within Gross’s framework, the subject of regulation is the individual: there is an actor who sets the goal, chooses the strategy, and implements it. Culture influences the direction in which this process unfolds, but it does not change the subject of the process. Emotion regulation, like emotion itself, is conceptualized as a process that takes place within the individual.

The concept of collective emotion regulation partially challenges this assumption. Research by Rimé et al. (2020) on emotional sharing reveals that narrating emotional experiences to others serves a regulatory function. Studies compiled by Von Scheve and Salmela (2014) document regulatory processes occurring at the group level. However, in these approaches as well, collective regulation is positioned as an extension of individual regulation occurring in a social environment: the individual shares their emotion, the group processes this emotion, and the individual finds relief. The starting and ending points of regulation are again the individual.

However, the African contexts examined present a different structure. Dzokoto et al.'s (2018) study of 7,015 Akan proverbs demonstrates that these proverbs normatively teach four basic regulation strategies within the framework of Gross's (2015) model. However, this normative transmission differs from an individual skill-acquisition process: the proverbs function as a collective memory repository, and the regulation strategy is applied by the community, on behalf of the community. Similarly, purification rituals, funeral practices, and collective mourning ceremonies perform the regulation function through ritual participation rather than individual cognitive processes (Hove et al., 2024; Turner, 2020). This distinction shows that ritualistic-performative emotion production, the second component of the proposed model, is also a regulation mechanism. Emotion regulation is not an individual skill but a collective achievement within the framework of relational ontology.

### 2.7 Beyond psychology: non-individual approaches to emotion

The theories examined so far constitute the mainstream frameworks of the psychology of emotion. However, objections to the individual positioning of emotion do not come solely from within psychology. Philosophy, sociology, and anthropology have long examined the collective and relational dimensions of emotion and questioned the individualistic assumption from different perspectives. Below, these approaches are evaluated in terms of both their contributions to the proposed model and the limitations they maintain.

First, Gergen's (2009) concept of relational being offers one of the most radical philosophical expressions of this objection. It argues that mental life and emotions are based not on an isolated individual but on collective action (co-action) and relationships. Following a similar line, the extended affectivity hypothesis suggests that emotions do not end at the biological boundaries of the individual (Colombetti and Roberts, 2015), while Krueger's (2014) approach of collective extended affect suggests that emotions can emerge as a structural feature belonging to a group. Slaby's (2014) concept of phenomenal coupling shows that individual experience is directly shaped by external expressions such as crowds or atmospheres. The overlap between these frameworks and Ubuntu is evident. This literature is valuable in two respects regarding the proposed model. First, Gergen’s framework offers the closest philosophical ground to Ubuntu ontology and demonstrates that relational existence is a universally defensible position, not unique to the African context. Second, the concept of phenomenal matching proposes a mechanism that can be directly used to explain how emotion is transmitted in ritual contexts. However, their starting point is individual affect, and the expansion metaphor itself presupposes a center, implying that what is expanding has a source. Furthermore, while these theories consider inanimate objects such as musical instruments or technological devices as the constitutive mediators of emotion (Colombetti and Roberts, 2015), Ubuntu addresses relationality in a moral dimension along the axes of human-human, human-community, and human-ancestor.

Social constructivist theories remove emotions from the realm of biological data and position them as products of culture and society (Aranguren, 2017). Harré's (1986) emotionology approach states that emotions are constructed within the grammar of the local language’s correct use of emotion words; Averill (1980) defines emotions as temporary social roles shaped around the functional needs of the social system. At this point, Harré’s emphasis on linguistic grammar offers a functional framework for explaining why the local concepts of emotion examined in this study cannot be translated into Western categories; concepts like *nagaa* or safuu are not merely different words, but different grammars of usage. However, within these frameworks, what is constructed is the individual’s emotional experience; the individual actor is the one who assumes the role and applies the grammar. Classical constructivism’s reduction of emotion to linguistic rules also excludes pre-linguistic and extra-linguistic forms of emotional belonging.

The sociology of emotion adds a different layer to the discussion, one that locates emotion within the structures of social interaction rather than within individual minds. Collins' (2004) theory of interaction ritual chains argues that focused interactions generate collective effervescence and a sense of belonging, which in turn creates emotional energy in individuals. This Durkheimian lineage is directly linked to the ritualistic-performative component of the proposed model. A parallel emphasis on social structure appears in Kemper's (1990) claim that emotions stem from power and status balances, and in Hochschild's (1983) concepts of emotional labor and feeling rules, which demonstrate how social expectations govern emotional expression, a mechanism that coincides with the normative teaching of emotion in Akan proverbs. The contribution of these theories to the proposed model is direct. Collins’ definition of ritual intensity as a measurable variable provides a usable tool for operationalizing the ritualistic-performative component of the model. Besides, Hochschild’s concept of feeling rules, beyond overlapping with the normative teaching of emotion in Akan proverbs, provides the necessary conceptual ground for a critical analysis of which emotions are considered legitimate. However, in these theories, collective processes produce individual outcomes: the collective is the cause, the individual is the effect. Another point of divergence is that Kemper’s framework places power and status balances at the foundation of emotion; this work does not deny the existence of power relations, but it grounds emotion not in hierarchical positioning but in relational belonging. Evolutionary and anthropological approaches broaden the discussion once again. Evolutionary sociology theories argue that the human brain has restructured itself to solve problems of social organization, creating deep sociality (Turner, 2000); this concept provides strong support for the proposed model, as it suggests that relationality may be a species-specific construct rather than merely a cultural preference. This is largely what prevents the Ubuntu ontology from being reduced to a claim of cultural relativism. Beatty (2014), on the other hand, suggests that emotion can be understood through a narrative approach where context, cultural model, and individual biography are intertwined. However, in the evolutionary framework, relationality is considered an adaptation located within the individual, while in Beatty’s perspective, me-focus is central.

Ecological systems theory adds a developmental dimension to the discussion. Bronfenbrenner (1979) considers individual development within a complex system extending from the microsystem to the macrosystem, demonstrating that family, community, cultural values, and historical conditions shape individual experience. This framework complements a significant shortcoming of the proposed model: Ubuntu ontology defines the relational basis of emotion, but it does not offer a developmental mechanism explaining how this basis is transmitted across generations. Bronfenbrenner’s layered structure provides a fruitful tool for conceptualizing the levels at which ritual participation and intergenerational transmission operate. However, in the model, systems are designed as rings surrounding the individual; the developing individual is at the center of the schema.

These theoretical families challenge the individualistic assumption of emotion research in different ways and form a significant part of the theoretical foundation of the proposed model. Indeed, all four components of the model have been shaped in dialogue with this literature. Nevertheless, a common structure is observed in all of them. Emotion originates from the individual and moves outward; it expands, is shared, regulated, or socially conditioned. What expands, is shared, or regulated ultimately remains an individual experience. The move proposed by the Ubuntu perspective is to reverse the direction of this movement: *“Emotion does not expand from the individual to the relationship; it individualizes from the relationship to the individual.”*

### 2.8 Ubuntu philosophy, collective emotion theory, and beyond individualist ontology

The common assumption of the four theories examined above is that the experiencer of emotion is an individual subject. Ubuntu philosophy, however, rejects this assumption at the ontological level. According to Tutu (2004), Ubuntu is a radically alternative understanding of existence against the Western Cartesian individualist ontology. Metz (2007) systematically addressed how Ubuntu can be conceptualized within moral philosophy. Similarly, Menkiti (1984) argued that in the African tradition, a person cannot exist independently of the community, and that personality is a status gained through social participation and moral responsibility. Wiredu (1996), on the other hand, demonstrated how this ontological framework fundamentally differs from Western individualism within the analytic philosophy tradition.

The literature on collective emotion in psychology empirically supports this philosophical argument. Hatfield et al.'s (1993) theory of emotional contagion posits that emotions spread automatically among individuals. Von Scheve and Salmela’s (2014) compilation of collective emotions, however, reveals that emotional structures emerging at the group level cannot be reduced to individual sums. Nevertheless, all these studies position the collective process as a “super-individual” phenomenon arising from the interaction of individual subjects. The Ubuntu ontology, the central argument of this study, proposes a more fundamental break. According to this approach, the relational network does not derive from individual subjects; rather, individual subjects derive from it.

Markus and Kitayama's (1991) “interdependent self” model positions the individual’s self within social relationships. However, the individual’s existential ground remains individual, and relationality is added to the individual as a quality. In the Ubuntu ontology, individual existence is positioned as a derivative of the relational network. De Jaegher and Di Paolo's (2007) participatory sense-making theory serves as a bridge at this point: meaning is the product of collective participation rather than individual cognitive processing. Van Kleef's (2009) EASI model and Keltner et al.'s (2022) study offer partial support to the relational perspective by suggesting that emotions carry social information. Rimé et al.'s (2020) findings show that the social sharing of emotions enhances social integration by creating Durkheimian “collective enthusiasm.” In the context of decolonial epistemology, Mudimbe's (1988) study demonstrates how Africa was “invented” by Western categories. In this study, decolonial epistemology is understood not as a binary opposition constructed as “the West versus the local,” but as a holistic critical framework that makes visible all power relations (including colonial and local ones), regardless of their origin. At this point, it should be clear that Ubuntu is used not as a normative ideal but as an analytical lens. Table 1 summarizes this ontological distinction, showing that Ubuntu and Western individualist frameworks differ not merely in cultural content but in the experiencing unit of emotion, its boundaries, its regulation, and its moral basis.

**Table 1:** Ubuntu and Western individualistic conceptions of emotion: a comparative framework.

Dimension	Ubuntu philosophy	Western individualist philosophy
Relational nature	Emotions connect people; feeling is a form of being with others.	Emotions separate people; feeling marks the boundaries of the self.
Locus of experience	Joy and sorrow belong to the community; the experiencing unit is the relational network.	Emotions reside in the individual mind and body; the person is the unit that experiences them.
Ontological priority	The relational network precedes the individual; individuals arise from relationships.	The individual comes before the relationship; relationships are formed between pre-existing individuals.
Source and boundary	A person’s inner world is constructed by others; there is no clear inside/outside boundary.	Emotions begin and end in a single person; the body is the boundary.
Regulation	Emotion regulation is a collective and ritualistic achievement.	Each person is responsible for managing and owning their own reactions.
Moral dimension	Emotional appropriateness is an obligation to the community and the cosmological order.	Emotional authenticity is a matter of individual self-expression.
Appraisal criteria	Ancestral relations, cosmic balance, social harmony	Individual goal alignment, personal well-being

## 3 Methodology

This study is designed as a “critical review” within the framework of Grant and Booth's (2009) typology of reviews. A critical review is a methodological approach that integrates a systematic literature review with theoretical analysis and critical evaluation. In this study, the systematic review process was structured to avoid reliance on random or selective sources. However, the primary aim here is not to statistically synthesize empirical findings, but to theoretically evaluate the explanatory capacity of the four emotions theory in the context of small-scale traditional African communities.

The combined use of PRISMA and critical review methodologies introduces methodological tension, which needs to be explicitly addressed. PRISMA prioritizes systematic sample selection and replicability, while critical review centers on the researcher’s interpretive judgment. This study uses the PRISMA 2020 guidelines (Page et al., 2021) not as a meta-analysis protocol but solely to ensure transparency in the search process. Inclusion decisions were made based on four predefined criteria and recorded along with their justifications. It is acknowledged that this procedure does not completely eliminate interpretive bias; however, it increases the verifiability of the analytical process.

Data were searched using the Web of Science, Scopus, and PsycINFO databases. The search was conducted between August 15 and November 15, 2025. The following search terms were employed: “African identity and emotion”, “African rituals”, “African tribal culture”, “African-centered psychology”, “emotion display rules”, “emotional enculturation”, “ethnopsychology and emotion”, “indigenous psychology and emotion”, “local emotion concept”, “rituals and emotions”, “small scale African societies”, “social norms and emotion”, “tribal culture and emotion.” Inclusion criteria were publication in an English-language peer-reviewed journal; direct focus on small-scale African traditional communities or tribal cultures; addressing the experience of emotion, emotion regulation, or the cultural-social construction of emotion in African communities; full-text access; and publication after 1990.

Initially, 2,625 records were identified; after eliminating 514 duplicate records, 2,111 studies were screened. The full texts of 172 studies were reviewed, and 57 studies were included in the analysis according to the inclusion criteria (see Figure 1). In terms of geographical representation, approximately 35% of the 57 included studies are from West Africa (primarily Ghana and Nigeria), 28% from East Africa (Ethiopia and Kenya), 22% from Southern Africa (South Africa and Zimbabwe), and 15% from Central Africa and multi-regional studies. This distribution demonstrates that the term “Africa” carries the risk of geographical and cultural homogenization. This study manages this risk at two levels. Firstly, at the theoretical level, the Ubuntu philosophy is used not as a meta-framework speaking on behalf of “all African communities,” but as an analytical lens rooted in the Bantu language family and exhibiting documented overlaps with the communities studied. Methodological differences are kept visible throughout the analysis, and examples of the model’s components are, as much as possible, supported by region-specific sources.

**Figure 1:**
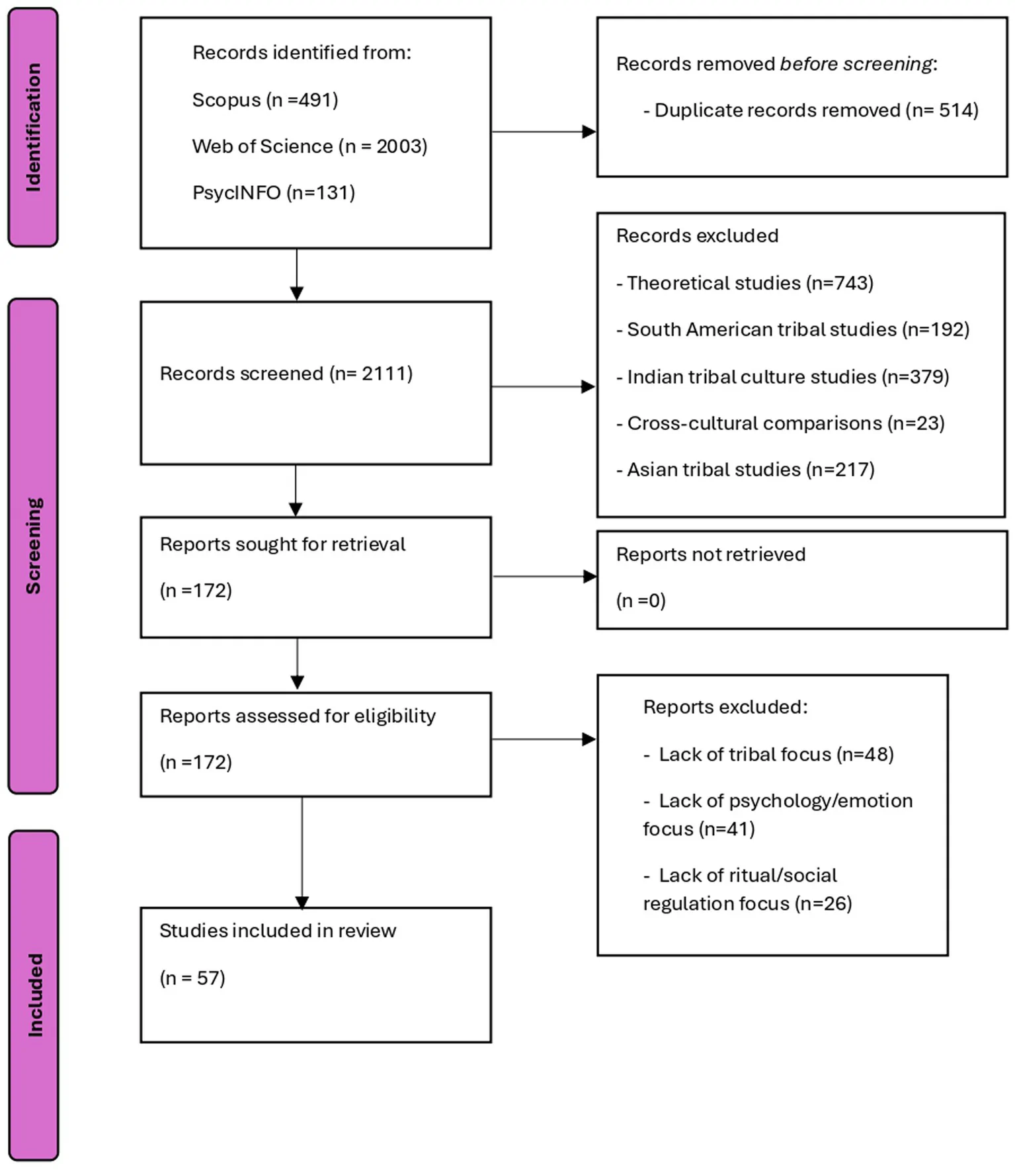
PRISMA flow diagram.

The Rayyan AI program was used for the systematic review process. The screening process was conducted by a single researcher, and a randomly selected 20% subsample (*n* = 34 studies) was evaluated by a second independent coder to verify reliability. Inter-coder reliability was assessed using Cohen’s kappa. The kappa coefficient is a statistical method used to determine the degree of agreement between two observers. The kappa test, which corrects for chance agreement between observers, is considered a stronger measure than agreement expressed as a percentage (Kılıç, 2015). In this study, Cohen’s κ = 0.81 (*p* < 0.001) was found in the inclusion/exclusion decisions. This value corresponds to the threshold of almost perfect agreement.

The studies included in the review were analyzed within the framework of four criteria: (1) competence to encompass culture-specific emotion categories, (2) capacity to explain ritualistic emotion production, (3) power to model collective and relational emotion processes, and (4) degree of inclusion of cosmological and spiritual dimensions. These criteria were inductively derived from the study’s research questions and form the analytical framework for evaluating theoretical explanatory power. The full list of 57 studies included in the review, the communities studied, and the emotional patterns examined, is presented in Supplementary material.

## 4 Results and discussion

### 4.1 The ontological framework of emotion in small-scale traditional African communities (RQ1)

#### 4.1.1 Relational ontology and Ubuntu

The vast majority of the studies examined show that emotions are conceptualized in these communities not as an individual inner experience, but as a dynamic part of collective existence. This finding qualitatively differs from the “culture influences emotion” argument in the existing emotion literature. Accordingly, in these communities, culture does not shape emotion from the outside; emotion already arises on a relational basis.

The Ubuntu philosophy’s statement “A person is a person through other people” expresses this ontological framework most intensely (Dwadwa-Henda et al., 2025). As discussed in Section 2.8, Ubuntu’s distinctive contribution lies in positioning individual existence as a derivative of the relational network rather than as a self situated within relationships.

The Ga people’s concept of “Dzɔɔmə” offers a concrete example of this relational ontology. Directly linking individual well-being to the well-being of society and the environment, this concept positions emotional well-being as a collective necessity (Ammah et al., 2024). For example, in Akan culture, shame is considered a socially disseminated experience rather than an individual one (Dzokoto et al., 2018). Similarly, the prohibition of expressing pain in ritual contexts according to the “Safuu” ethic of the Borana Oromo people, and the concept of “Isinyama” in Tsonga and Xhosa cultures, all demonstrate that emotion functions as an indicator of relational and cosmological balance rather than individual psychological states (Duressa, 2022; Hove et al., 2024).

#### 4.1.2 Linguistic and epistemological foundations of emotion categories

Although local concepts of emotion in these communities show partial overlap with Western categories, they mostly carry semantic dimensions that cannot be reduced to these categories (see Table 2). As Wierzbicka (1999) points out, emotion words cannot be translated directly across cultures; each language carries its own unique emotion ontology. The empirical findings of Gendron et al. (2012) also support this view.

**Table 2:** Selected local concepts of emotion: cultural meaning and unexplained dimensions.

Concept	Culture	Cultural meaning	Wester-equivalence	Unexplained dimensions
Nagaa	Borana Oromo	A holistic sense of peace and well-being is defined by the harmony between God, humans, and nature.	“Happiness”/“Contentment”	Encompasses cosmological and ecological balance; cannot be reduced to individual well-being
Safuu	Borana Oromo	A moral/cosmic norm regulating the relationship between humans, God, and nature.	“Shame”/“Moral emotion”	Simultaneously emotional, normative, and spiritual; cannot be placed in a single category.
Umbilini	IsiXhosa	An intuitive sense of unease felt in the internal organs; a source of pre-reflective knowledge.	“Anxiety”/“Intuition”	A somatic-spiritual form of knowledge; standard anxiety scales miss this dimension entirely

For the complete list of local emotion concepts, see Supplementary Table S2.

This conceptual differentiation is not only a theoretical problem but also raises a critical validity issue at the measurement level. For example, tools such as PANAS (Watson et al., 1988) are based on the assumption of a “positive/negative” duality. However, concepts such as umbilini or safuu cannot be placed within this duality. The bodily-spiritual information dimension of umbilini, felt in the internal organs, does not fit into either the positive or negative pole. According to Mauss and Robinson (2009), the physiological, behavioral, and subjective components of emotion measurement often operate independently of one another, and this inconsistency is exacerbated in cultural contexts.

#### 4.1.3 The role of cultural norms in regulating emotion and the critical dimension

The study by Dzokoto et al. (2018) on 7,015 Akan proverbs shows that these proverbs normatively teach four basic emotion regulation strategies within the framework of Gross's (2015) model. As Vishkin and Tamir (2023) emphasize, norms target not only the display of emotion but also the emotion itself. However, an analytical balance is necessary when emphasizing the functional dimension of cultural norms. Individuals tend to respond to standard self-report tools in ways that reflect the normative ideal rather than their emotional reality. This constitutes a structural form of social desirability bias stemming from cultural norms. The studies examined also show that the same normative systems, in some contexts, transform into mechanisms that oppress the individual (especially women and marginalized groups). The imposition of pain-free behavior in adolescents during traditional male circumcision rituals (Siweya and Sodi, 2018) and the association of female genital manipulation with social acceptance and identity construction (Omigbodun et al., 2022) are concrete examples of this repressive function. When analyzing cultural norms as functional structures that regulate emotion, the question of “whose emotion is defined as legitimate and whose emotion should be suppressed” must also be asked.

### 4.2 Theoretical analysis: explanatory capacity and limitations of the four theories (RQ2)

The explanatory capacity assessment of the four theories was conducted not only with the question “Does it encompass cultural context?” but also with the more fundamental question “Does this theory question the individual nature of the subject experiencing emotion?” Table 3 summarizes the comparative analysis centered on this question. The four theories are examined here in terms of their explanatory capacity in the African context; the non-psychological approaches discussed in Section 2.7 are not included in this comparative table, as they contribute to the ontological argument rather than having been applied to this specific context.

**Table 3:** Comparative explanatory analysis of four theories.

Theory	Supported aspects	Limitations	Ontological distance	Explanatory power
Basic Emotions Theory (Ekman, 1992)	Recurrence of fear, anger, anxiety, and grief categories across different communities	Ubuntu-centered emotions and ritualistic production fall outside the scope; biological essentialism cannot account for collective emotion.	Highest- the individual biological subject is presupposed	Low
Cognitive Appraisal Theories (Ellsworth and Scherer, 2003; Lazarus and Smith, 1988)	Relationship between appraisal schemas of threat, betrayal, and loss of prestige and emotion production; “structural distrust” finding in COVID-19 vaccine hesitancy	Focus on individual goal congruence excludes collective cognition; cosmological appraisal falls outside the scope.	High- the appraising subject is an individual	Moderate
Constructionist Theory of Emotion (Barrett, 2017)	Culture-specific concepts and ritualistic emotion production align with theoretical predictions	The individual brain and body remain the subject of construction; historical trauma systematically excluded	Moderate- acknowledges cultural construction, but the subject remains individual	High (with limitations)
Cultural Norm Theory (Boiger and Mesquita, 2012)	High compatibility with relational emotions, Ubuntu ethics, and collective identity regulation; social signaling function of positive emotions (Joshanloo and Weijers, 2025)	Limited in spiritual-cosmological emotion systems; insufficient in contexts where norms are instrumentalized for patriarchal structures and structural violence; the question of whose emotions are deemed legitimate remains unanswered	Lowest- centers relational process	High (with limitations)

#### 4.2.1 Basic emotions theory: low explanatory power

The reporting of categories such as fear, anger, and anxiety as recurring experiences in different communities partially supports the assumption of a common emotional repertoire. However, there are serious limitations at two levels: empirical and ontological.

The empirical limitation is that rituals in which the expression of pain is forbidden due to “Safuu” ethics directly refute the assumption that emotion is spontaneously expressed through universal facial expressions. When the neuroscientific data from Jack et al. (2012) and the study findings from Crivelli et al. (2016) are considered together, the empirical basis for the universality of facial expressions appears quite fragile.

At the ontological level, concepts such as pride, honor, collective enthusiasm, and nagaa fall outside Ekman’s classification, yet still assume central emotional functions in these communities. This should not be considered a coincidental deficiency. Since Basic Emotions Theory necessarily presupposes the individual biological subject, it cannot, by definition, include emotion categories that arise on a relational basis within its conceptual framework.

#### 4.2.2 Cognitive appraisal theories: moderate explanatory Power

The findings support the relationship between appraisal schemas, such as threat, betrayal, loss of status, and the production of emotions (Cochrane, 2022; Stambach and Pesambili, 2025). The finding that appraisal processes are culturally constructed (e.g., the “structural insecurity” phenomenon in COVID-19 vaccine hesitancy) shows that the theory’s basic mechanism also operates in this context. However, collective and cosmological appraisal mechanisms are excluded from this theory. The evaluation of disease as “the wrath of the ancestors” in a tribal context or the collective anxiety in rain rituals (Van Beek, 2025) cannot be explained within the framework of individual goal alignment. Since Cognitive Appraisal Theories position the evaluating subject individually, they must treat the question “What does it mean for us?” as a derivative of the question “What does it mean for me?.” In the Ubuntu ontology, however, collective evaluation does not derive from individual evaluation; rather, individual evaluation arises from the collective ground.

#### 4.2.3 Constructivist theory of emotion: high explanatory power (with limitations)

Findings on culture-specific concepts, ritualistic emotion production, and linguistic framing strongly align with the theory’s predictions. Turner's (2020) analysis of dīwān rituals and Yepthomi et al.’s (2025) study of Naga tribes provide strong empirical support for the view that emotion is constructed through cultural concepts.

The main limitation of constructivist theory is that while it strongly explains cultural construction, it continues to center the individual brain and body as the subject of construction. Barrett’s concept of “core affect” suggests a process that transcends the individual brain, emphasizing that bodily sensations gain meaning within a social context. While integrating this concept with Ubuntu ontology is theoretically possible, Barrett’s model, in its current form, has not taken this step. In addition, the structural impact of historical trauma and colonial legacy on emotion patterns is systematically excluded (Geissler and Prince, 2020). This structural effect is also reflected at the measurement level. Emotional responses stemming from the legacy of colonialism can be miscoded as pathology using standard tools.

#### 4.2.4 Cultural norm theory: high explanatory power (with limitations)

Findings on relational emotions, normative regulation, and Ubuntu ethics strongly align with the theory’s core predictions. Joshanloo and Weijers' (2025) finding of “the social signaling function of positive emotions” reinforces this alignment.

Of the four theories, Cultural Norm Theory is the approach that is the least distant from individualistic ontology because it positions emotion as a relational process from the outset. Despite the insights this theory provides, two significant limitations persist. Firstly, the frameworks surrounding spiritual and cosmological emotion systems are insufficiently explored, leaving a gap in the understanding of how these dimensions affect human experience. Secondly, the theory falls short in addressing scenarios where cultural norms are manipulated to uphold patriarchal structures and perpetuate structural violence, making it difficult to analyze these issues from a critical standpoint. This oversight limits the potential for a comprehensive critique of the intersections between emotion, power, and societal norms. The proposed Ubuntu-based model aims precisely to achieve this expansion.

### 4.3 Ubuntu-oriented four-component model (RQ-3)

The review findings show that the common limitation of existing emotion theories is not cultural content, but on the ontological ground. The proposed model addresses this inquiry through four components (see Table 4).

**Table 4:** Ubuntu-based emotion model: components, mechanisms, and testable propositions.

Model component	Definition and mechanism	Testable propositions
1. Relational Ontology (Ubuntu Principle)	Emotion is a product of the relational network, not of the individual’s internal state. Appraisal shifts from individual goals to collective goals. Distinction from collective emotion theories (Von Scheve and Salmela, 2014): the relational network is the ground from which individuals emerge, not a supra-individual output.	P1: Collective identity threats will produce stronger emotional activation than individual identity threats. P2: Violations of emotion display rules in Ubuntu-centered communities will result in stronger social sanctions.
2. Ritualistic-Performative Emotion Production	Rituals are not merely arenas for the expression of emotion but are mechanisms of its production. Whitehouse's (2004) Modes of Religiosity Theory explains how ritual intensity generates cognitive-emotional synchronization.	P3: Intensity of ritual participation will be positively associated with emotion regulation capacity and levels of collective effervescence. P4: Ritual exclusion will produce more intense negative emotion compared to standard social exclusion manipulations.
3. Cosmological Appraisal Dimension	Cognitive appraisal extends beyond individual goal congruence; the ancestor-human relationship, cosmic balance, and spiritual obligation are also included as appraisal dimensions. McCauley and Lawson's (2002) cognitive theory of religion explains how cosmological beliefs shape cognitive processing.	P5: In individuals with high cosmological belief levels, emotional responses to negative life events will be coded through relational/spiritual disruption attribution. P6: Participation in purification rituals will yield more effective emotion regulation compared to cognitive reappraisal intervention alone.
4. Decolonial Epistemology	Local languages, proverbs, and ritual narratives are recognized as theoretical sources; the structural impact of colonial legacy on emotional patterns is incorporated into the model; power relations within local normative systems are analyzed concurrently. This component is not a “local versus Western” binary but a holistic critical framework encompassing all power relations.	P7: Activation of collective memory regarding colonial history will significantly differentiate current emotional responses compared to WEIRD samples. P8: Emotion measurement tools administered in local languages will demonstrate higher factor validity compared to translated Western scales.

According to the model’s theoretical status, each of these four components (relational ontology, ritualistic production, cosmological evaluation, and decolonial epistemology) is a concept that exists separately in literature. This model should be understood not as an original synthesis of the components, but as an integration framework that answers the question: What emotional phenomena can these components jointly explain that an individualistic ontology cannot structurally explain? The model’s original contribution should be sought not in the content of the components, but in their hierarchical and complementary structure and in the ontological grounding of this structure provided by Ubuntu ontology. The model’s distinguishing feature is that its components form a hierarchical and complementary structure, rather than being independent of each other: relational ontology is the fundamental basis of the theory (ontological layer); the ritualistic production mechanism (practical layer); cosmological evaluation is the cultural content of cognitive processes (cognitive layer); and decolonial epistemology is the meta-level principle framing the entire model.

The concept of a “relational network” should be understood not only as a philosophical meta-language but also as a structure that can be tested at the operational level. The psychological mechanisms corresponding to relational ontology can be sought at three levels. At the first level, social network analysis: Kashima et al.'s (2020) framework allows mapping of how emotional experience spreads from one individual to another within a community. At the second level, physiological synchronization: Whether the physiological indicators (heart rate, cortisol, skin conductivity) of group members are synchronized during ritual participation can test the bodily-level counterpart of the relational network. At the third level, collective emotion regulation capacity: The relationship between the intensity of ritual participation and individual emotion regulation capacity can be measured as a functional indicator of the relational network.

The theoretical validity of the proposed Ubuntu-based model is grounded in indirect empirical (proxy) data in the literature. In particular, the proposition (P1) that a threat to collective identity will produce stronger emotional activation than a threat to individual identity is supported by studies of Akan culture. Indeed, in these communities, the concept of “shame” is coded as a social stigma rather than an individual mistake and is seen as “worse than death,” revealing the sensitivity of the emotional system to damage to collective honor (Dzokoto et al., 2018). Similarly, the proposition (P8) that measurement tools in the local language will show higher validity is justified by findings that existing Western-centric scales fail to reduce holistic concepts such as *nagaa* or safuu to valence-arousal dimensions, thus missing the ontology of emotion. However, these propositions are not inductively derived findings but research orientations generated by the model; the indirect data above support their plausibility, though studies designed to test them remain necessary.

## 5 Conclusion and recommendations

### 5.1 Theoretical results

This study questioned the WEIRD bias in the psychology of emotion, not only at the sample level but also at the ontological level. The findings reveal five main conclusions.

First: In small-scale traditional African communities, emotions are conceptualized not as individual experiences shaped by culture but as collective phenomena arising within relational networks grounded in the Ubuntu philosophy.

Second: The explanatory capacity of the four theories is directly proportional to their distance from individualistic ontology. The Basic Emotions Theory shows low explanatory power because it most strongly assumes the individual biological subject. Cognitive Appraisal Theories are moderately explanatory due to collective expansion. Constructivist and Cultural Norm theories have high but insufficient explanatory power because their distance from individualistic ontology is relatively low.

Third: No single theory is sufficient on its own. The reason for this insufficiency is not only a lack of cultural content but also a structural ontological constraint.

Fourth: The proposed four-component Ubuntu-centric model differs from existing theories of collective emotion (Barsade, 2002; Von Scheve and Salmela, 2014) on this fundamental point. While existing theories position the collective process as a meta-individual phenomenon arising from the interaction of individual subjects, the Ubuntu model positions the relational network as the ground from which individual subjects are derived. This distinction is kept open to empirical research with the model’s eight testable propositions.

Fifth: Western-centric measurement tools in these contexts pose a problem not only of cultural validity but also of epistemological legitimacy. Concepts such as nagaa or safuu cannot be reduced to valence-arousal dimensions; emotional responses stemming from the legacy of colonialism can be miscoded as pathology by standard scales.

### 5.2 Limitations

This study presents both notable contributions to the field and identifiable limitations. The main limitations of this study are as follows: First, the included studies are English-language peer-reviewed journal publications; access to resources in local languages was not possible. Second, the theoretical framework is structured around four main theories; dimensional models (Russell, 1980), the SPAARS model, and phenomenological approaches were not systematically included. Third, the proposed model has not yet been empirically tested. Fourth, hybrid emotional identity patterns emerging amid globalization, digitalization, and urbanization are outside the scope of this study and constitute a critical agenda item for future research. Finally, this review is geographically limited to small-scale traditional communities in Africa. However, similar relational-ontological frameworks and ritualistic emotion-production mechanisms have been documented among indigenous communities in Asia, South Asia, and South America. The generalizability of the proposed Ubuntu-based model to these contexts remains to be examined.

### 5.3 Recommendations

Theoretical recommendations: It is recommended that appraisal theories be expanded to include collective-cosmological dimensions, particularly by bridging the gap between Scherer's (2001) component process model and cosmological evaluation; and that constructivist and cultural norm approaches be systematically revised to encompass the colonial legacy and historical trauma. At a more fundamental level, emotion psychology needs to question the assumption of an individualistic ontology and bring relational ontology frameworks to the theoretical center. Additionally, future research should investigate whether the four-component model proposed here extends to non-African indigenous communities. Comparative studies involving Amazonian tribes, South Asian tribal communities, and Pacific Islander societies would be particularly valuable in determining whether relational ontology constitutes a broader cross-cultural alternative to individualist emotion theories, or whether it is specific to Ubuntu-centered African traditions.

Methodological recommendations: Development of psychometric tools based on local concepts; testing the P8 proposition; and determining whether measurement tools applied in the local language show higher factor validity than translated Western scales constitute a priority research agenda. It is recommended that Mauss and Robinson's (2009) multi-component measurement logic be restructured with culture-specific content.

Clinical and applied recommendations: In clinical interventions, the most direct practical reflection of the comparison presented in Table 1 is seen in the field of emotion regulation. While Western-centric psychotherapy models position emotion regulation as an individual skill, in the contexts examined, this process is a collective and ritualistic achievement. When this distinction is ignored, individually focused interventions can have a counterproductive effect by disconnecting the client from their support network. Therefore, it is recommended that emotions be addressed within a cultural-relational context rather than as individual pathology in clinical interventions; that family and community-participatory approaches be prioritized; and that local language and narrative therapy methods be used in the therapeutic process (Gqweta, 2025). Similarly, defining well-being solely as an individual outcome renders community-based support systems invisible; indicators such as ritual participation and intergenerational belonging warrant inclusion in mental health policy assessments.

## Data Availability

The original contributions presented in the study are included in the article/Supplementary material, further inquiries can be directed to the corresponding author.
