# Supplementary material for: The relational ground of emotion: an Ubuntu-based critique of individualist ontology in theories of emotion
Source: Front Psychol. 2026 Sep 14;17:1926813. doi: 10.3389/fpsyg.2026.1926813 (PMC13617129; doi:10.3389/fpsyg.2026.1926813)
Supplement: Supplementary file 2 [file Table_1.DOCX]

**Supplementary Materials**

**Table S1.** List of 57 included studies with characteristics

| No | Author(s) | Community/Culture | Emotions |
| --- | --- | --- | --- |
| 1 | Ammah et al. (2024) | Ghana, Ga people, Akan/Ashanti predominantly | Social cohesion, Peace, Well-being |
| 2 | Appiah (2024) | Ghana and Africa generally (especially Akan proverbs) | Well-being, Flourishing, Positive relationships, Kindness/Empathy, Gratitude, Honesty/Integrity, Patience/Humility |
| 3 | Atabongwoung et al. (2023) | South Africa, African Religion and Pentecostalism followers (Nguni people) | Interfaith Consciousness, Reconciliation, Common Good |
| 4 | Bhuda et al. (2023) | South Africa, Batswana | Grief and Consolation |
| 5 | Boyette (2025) | Congo, Aka, and Ngandu | Risk perception in children, Anxiety (in Western intensive parenting culture), Respect |
| 6 | Campbell (2018) | Northern Nepal, Tamang-speaking communities | Moral economy, Subsistence Anxieties, Relational tensions, Trauma |
| 7 | Charwi (2025) | African indigenous religions (Yoruba, Christianity, Islam) | Resilience, Unity and solidarity, Ethical values, Anger, Lamentation, Cursing, Remorse |
| 8 | de Jong (2015) | South Africa (KwaZulu-Natal, Limpopo) | Compassion, Anger |
| 9 | Dessì (2020) | South Africa, Soka Gakkai International (SGI) members | Empowerment, Overcoming fear and weakness, Anger management |
| 10 | Dessì (2022) | South Africa, East Asian Buddhist groups (Dharma Center, SGI, Foguangshan) | Stress, Anxiety, Anger, Balance |
| 11 | Dlamini (2025) | South Africa (isiZulu language and Isolezwe readers) | Defeat, Disappointment, Anger, Betrayal, Shock, Disgust, Amusement |
| 12 | Duressa (2022) | Southern Ethiopia, Borana Oromo people | Gratitude (Galata), Peace (Nagaa), Prohibition of expressing pain (Safuu ethics) |
| 13 | Dwadwa-Henda et al. (2025) | South Africa, AmaBomvane (a subgroup of amaXhosa) | Stigma and Exclusion, Inner Strength and Belonging |
| 14 | Dzokoto et al. (2018) | Ghana, Akan society | Pain/Suffering, Fear, Shame/Disgrace, Anger, Pride (unwanted) |
| 15 | Ema et al. (2023) | Nigeria, Obudu community, Cross River State | Pride, Goodwill |
| 16 | Esoh and Kaunda (2020) | Northwest Cameroon, Obang culture | Libation emotions, Respect, Devotion, Unease/Fear (of ancestral wrath) |
| 17 | Geissler and Prince (2020) | Western Kenya communities | Traumatic memories, Grief, Anger, Unease, Dignity |
| 18 | Gqweta (2025) | South Africa, Xhosa people (amaXhosa, isiXhosa language) | Pride, Sense of belonging, Devotion, Emotional expression, Traumatic silence |
| 19 | Havelka (2022) | Africa, practitioners of the Gabonese Bwiti religion, Gabon | Self-discovery, Insight, Spiritual growth, Risk/Fear |
| 20 | Hosny et al. (2023) | North Africa (Somalia, Cairo) and the Middle East | Anxiety, Fear, Hopelessness, Melancholy |
| 21 | Hove et al. (2024) | South African tribes | Devotion, Disrespect, Shame, Blame |
| 22 | Hove (2024) | South Africa, Limpopo, Collins Chabane Municipality (Tsonga, Venda, Pedi peoples) | Respect and Dignity, Grief and Healing |
| 23 | Ishemo (2002) | Africa, Cameroon, Sociedad Secreta Abakua | Identity insecurity, Grief, Collective Honor, Fear |
| 24 | Kahissay et al. (2020) | Northeastern Ethiopia, Tehuledere people | Emotional wellness, Madness, Fear, and Anxiety (from illness causes), Spirituality |
| 25 | Kpanake (2018) | Africa (Nigeria, Akan, Ghana, etc.) | Ubuntu, Devotion, Pain, Respect, Psychological well-being, Fear, and Anxiety (from evil spirits) |
| 26 | Lebaka (2019) | South Africa, Limpopo, Bapedi people | Respect, Fear and Worry, Hatred, Hope |
| 27 | Lebaka (2020) | South Africa, Limpopo, Bapedi people | Respect, Devotion, Joy, Gratitude |
| 28 | Lotsu (2025) | Ghana (Funeral ritual) | Mourning, Grief, Sorrow, Pain, Joy, Respect, Sadness, Lamentation |
| 29 | Luka and Martin (2025) | Nigeria, Bajju people | Collective effervescence, Hostility, Love, Unity, Respect |
| 30 | Madlome (2022) | South Africa (Tsonga, Vatsonga) | Fear, Loneliness, Loss of identity (nicknaming), Unity, Compassion |
| 31 | Memela et al. (2025) | South Africa, Makhanda | Sense of belonging, Security, Respect, Gratitude |
| 32 | Michael and Mensah (2025) | Nigeria, Nsirimo (Igbo) people | Gratitude, Humility, Honor, Dignity, Anger |
| 33 | Mire (2015) | Somali women | Fertility anxiety (Wagar), Spiritual reassurance, Joy, Disappointment, Jealousy |
| 34 | Mire (2016) | Somali women | Fertility anxiety (Tiire), Hope, Hopelessness, Emotional reassurance, Anxiety, Anger, Sadness |
| 35 | Mthembu (2020) | South Africa, KwaMashu (Black African youth experiences) | Security, Respect, Responsibility, Hope, Devotion, Gratitude, Pride |
| 36 | Mukosi et al. (2025) | South Africa, Vhavenda people (Names) | Trust, Grief, Shame, Glorification |
| 37 | Ndetei et al. (2023) | African perspective (general) | Boredom |
| 38 | Ngcobo (2020) | South Africa, AmaZulu (Zulu people) | Respect, Pain, Hope, Privacy, Pride, Togetherness |
| 39 | Ngubane and De Gama (2024) | South Africa, KwaZulu-Natal | Aggression, Hallucinations, Depression, Peace, Helplessness, Fear |
| 40 | Ohaja and Anyim (2021) | African perspectives (Cameroon, Ghana, Madagascar, Kenya, Nigeria, Mali, Tanzania, Uganda) | Joy and Happiness, Compassion, Anxiety, and Fear (from supernatural threats) |
| 41 | Omigbodun et al. (2022) | Southeastern Nigeria, Izzi people | Fear of social rejection, Fear of negative stigma, Embarrassment, Joy, Shame |
| 42 | Opata and Asogwa (2017) | Southeastern Nigeria, Nsukka Igbo people | Spiritual respect and reverence, Honor and social prestige |
| 43 | Oyelade and Omobowale (2022) | Southwestern Nigeria, Yoruba Pentecostal believers | Fear and Anxiety (from spiritual evil), Existential anxiety, Anger, Courage, Doubt |
| 44 | Petrollino (2025) | Southwestern Ethiopia | Group cohesion and belonging, Joy, Responsibility, Relief |
| 45 | Phalatsi-Shilubana et al. (2024) | South Africa, Zulu King | Sacredness, Unity, Pride, Awe |
| 46 | Rasmussen (2022) | Nigeria, Tuareg (Kel Tamajaq) people | Luck, Love, Happiness, Anger, Jealousy, Doubt, and Distrust |
| 47 | Ratele and Rustin (2023) | South African women | Happiness, Satisfaction, Contentment, Collective joy |
| 48 | Reyes-García et al. (2020) | Southern and Central Africa, Tsimane' (Bolivia) and Baka (Cameroon) hunter-gatherer societies | Gender role-related emotional states (Constraints/Facilitators), Courage, Motivation, Cooperation |
| 49 | Roba (2022) | Southern Ethiopia, Guji community | Fear and Anxiety (regarding COVID-19), Helplessness, Disgust |
| 50 | Roos et al. (2014) | South Africa, Northwest Province, forcibly displaced elderly women | Security, Sense of belonging, Respect, Devotion |
| 51 | Singh and Bhagwan (2020) | South Africa (Zulu people, African spirituality) | Coping with trauma, Forgiveness, Respect, Anxiety |
| 52 | Siweya et al. (2018) | South Africa, Limpopo (Ngove Village, Circumcision) | Fearlessness, Aggression, Shame, Humiliation |
| 53 | Stambach and Pesambili (2025) | South African and Tanzanian communities | Political distrust, Fear, Suspicion, Threat, and Betrayal (vaccine hesitancy), Panic |
| 54 | Titi (2023) | South Africa | Distrust, Trauma related to sexual violence |
| 55 | Turner (2020) | Algeria, Diwān Mûsâ (Sufi/religious) community | Grief, Pain, Fear, Renewal, Hope, Resilience |
| 56 | van Beek (2025) | Religions across Africa (Nigeria, Kapsiki/Higi) | Hope and Anxiety, Fear (of rain failure), Respect |
| 57 | Wilson et al. (2018) | Ghana and South Africa | Hope, Well-being, Collective pride and responsibility |

**Table S2.** Local emotion concepts and their cultural meanings

| Author(s) | Local Concept | Cultural Meaning and Emotional Function |
| --- | --- | --- |
| Ammah et al. (2024) | *Ubuntu* | A continental ethical-emotional framework expressing interconnectedness, compassion, solidarity, and community-based affect. Moves emotion out of the individual sphere and regulates it as "relational moral responsibility." |
|  | *Kpodziemo* | A ritual of presenting the newborn to the community in Ga society. Establishes feelings of joy, collective pride, hope, and communal belonging; emotionally and morally binds the individual to the community from birth. |
|  | *Dzoomo* | The central prayer/benediction within KpodziemO. Contains wishes for the baby's health, happiness, relationships, and responsibilities. Sets the emotional codes of "the good life," centering hope, harmony, responsibility, and reciprocity. |
|  | *Wobole kutu wo kpe* | A communal integrity wish meaning "may our circle hold firm." A normative emotional principle preserving feelings of security, belonging, social harmony, and community. |
|  | *wodze bu wodze nu* | "May water rise from the well we dig" : a wish for communal labor to be fruitful. Structures feelings of hope, shared success, reciprocity, and collective satisfaction. |
| Charwi (2025) | *Oríkì Orí* | Praise chants in Yoruba tradition addressing one's "orí" (destiny/head/personal essence). Strengthens feelings of pride, honor, ancestral ties, and collective identity; links individual self-worth to the community. |
| Duressa (2022) | *Safuu* | A moral/cosmic norm regulating the human–God–nature relationship; the overarching framework determining feelings of respect, moderation, and awareness of limits. Classifies emotions as legitimate or illegitimate. |
|  | *dhibaayyuu* | A gratitude ritual performed after a crisis; transforms fear, uncertainty, and pain into collective thankfulness and trust. Strengthens feelings of hope and reconnection. |
|  | *nagaa* | A holistic state of peace and well-being defined by the harmony of God–human–nature; represents the ideal emotional atmosphere of the community. |
| Esoh and Kaunda (2020) | *Sar’h / Ezul* | Local names for libations offered to ancestors in Obang society; triggers feelings of reverence, gratitude, and awe-tinged respect. Keeps the emotional bond with ancestors alive. |
| Dlamini (2025) | *bahlonipheke* | "To appear respectful/honorable" in isiZulu; regulates the emotional norms of hlonipha culture (humility, shame, respect). Preserves the feeling of social honor. |
|  | *usaba* | "To fear" in isiZulu; an emotion that opens for debate the legitimacy of women's fear in gendered contexts. Makes visible the politicized nature of fear. |
| Dwadwa-Henda et al. (2025) | *Ubuntu* | A holistic emotion-ethics system emphasizing mutual interconnectedness, compassion, and solidarity among people. Reframes individual pain and shame in favor of the community. |
| Dessi (2020) | *kōsen-rufu* | A vision of global peace and justice in Nichiren Buddhism; organizes feelings of hope, solidarity, and collective struggle. Links personal suffering to social transformation. |
|  | *shakubuku* | A confrontational practice meaning "to teach by breaking false beliefs"; transforms anger and frustration into feelings of ethical struggle and courage. |
|  | *daimoku / Nam-myōhō-renge-kyō* | Rhythmic recitation of the Lotus Sutra's name; a vocal meditation that transforms fear, pain, and hopelessness into feelings of calm, empowerment, and hope. |
| Dzokoto et al. (2018) | *abofuo* | "Anger" in Akan; a powerful emotion that becomes destructive when uncontrolled. A normative emotion teaching the proper management of anger for social harmony. |
|  | *fεre (fere)* | "Shame" in Akan; one of the most severe emotional consequences of social face loss. Functions as a mechanism of social control. |
|  | *odɔ (odO)* | "Love" in Akan; defined on the basis of sacrifice, devotion, and forgiveness. A positive emotion that strengthens social harmony and relational responsibility. |
| Gqweta (2025) | *intliziyo yam ilihlwili* | "My heart is shattered"; describes intense emotional pain, loss, and hopelessness; allows the social recognition of inner suffering. |
|  | *intliziyo yam ibuhlungu* | "My heart aches"; expresses psychological pain through the metaphor of physical pain; grants "real pain" status to emotional suffering. |
|  | *ndinombilini* | Intuitive unease and anxiety felt around the abdomen; reflects an emotional ontology that does not separate the emotional from the bodily. |
|  | *umbilini* | A concept used for the internal organs/lumbar region and intuition/"inner feeling"; positions pre-reflective, non-cognitive affect as a primary source of knowledge. |
|  | *Andiziva kamnandi namhlanje ingathi kukhona into endizokuyiva* | The expression "I don't feel well today, as if something bad is about to happen"; articulates anticipatory anxiety and a sense of foreboding; frames anxiety as both individual and cosmic warning. |
|  | *ikati ilele eziko* | The proverb "the cat is sleeping by the hearth"; implies complacency in the face of approaching danger; carries a feeling of warning and mild anxiety through metaphor. |
| Hosny et al. (2023) | *welwel* | A cultural distress term describing persistent unease, inner constriction, and mournful mood following trauma; by naming the chronic anxiety–grief mixture it both normalizes it and legitimizes seeking help. |
|  | *zar / wadodo* | A narrative of spirit possession; enables the expression of suppressed anger, resentment, and helplessness — especially in women — as "spirit invasion"; reduces shame and moves emotions into a spiritual frame. |
|  | *hozn* | Melancholy, unease, anger |
|  | *majnun* | Unease, excessive anger, guilt |
|  | *buufi* | Anxiety symptoms, paranoia, obsession |
|  | *Marrora dilla* | Powerlessness, resentment and anger following someone's loss |
| Hove vd. (2024) | *idlozi* | Ancestral spirit/guardian spirit; associated with personality, destiny, and protection; simultaneously organizes feelings of respect, fear, gratitude, and belonging. |
| Hove (2024) | *isililo* | Symbolic mourning, lamentation |
|  | *isinyama* | Purification/cleansing, purging of evil contamination from the dead |
|  | *isithunzi* | Purification from evil spirits, spiritual cleansing |
| Kahissay et al. (2020) | *buda* | "Evil eye/harmful power" directed by jealous and malicious individuals; links illness and misfortune to envy, indirectly channels jealousy, and strengthens the social order (by controlling envy). |
|  | *wadaja* | A ritual of collective prayer, votive offering, and sharing; enables collective expression of fear, hope, guilt, and gratitude; transforms individual suffering into shared communal ritual. |
|  | *qolle / quteb* | Nature spirits associated with specific places; harmony or rupture with them affects one's health and emotional state; draws environmental and moral boundaries through fear and respect. |
| Kpanake (2018) | *ubuntu / umundu / bumuntu / vumuntu / nunhu / bomoto / gimuntu* | "Humanity/humanness" in various Bantu languages; expresses "becoming a full person" through compassion, solidarity, and mutual responsibility; ties personhood to a relational and emotional ontology. |
|  | *Bubedi bwa disu mbubedi bwa diulu* | "The sickness of the eye is the sickness of the nose"; makes affect the measure of personhood as a criterion for deepening social bonds. |
|  | *Wiase yi mu yedi no nnoboa* | "Life is a mutual help venture" (Akan); normalizes feelings of interdependence, solidarity, and community pride. |
|  | *oye onipa paa* | "He/she is truly human" (Akan); praises desirable emotional-moral qualities such as kindness, generosity, and responsibility; makes these qualities the criterion of "full humanity." |
|  | *onnye ’nipa* | "He/she cannot be counted as human" (Akan); frames emotional coldness, selfishness, or disrespectfulness as "loss of humanity"; serves as a powerful emotional/normative sanction. |
| Lebaka (2019) | *Dikeledi* | "Tears" — a meaningful name. Symbolizes that loss and difficult experiences are an emotional heritage that must not be forgotten; constructs an identity of grief and sorrow. Appended to the end of the name. |
|  | *Matshwenyego* | "Hardship/difficulty" : a meaningful name. Links individual life narratives to collective economic and political struggles, coding hardship as a normalized existential condition. |
|  | *Mahlogonolo* | "Good luck" : a meaningful name. Associated with bringing joy and success to the person's life. |
|  | *Kgotsofalo* | "Satisfaction/contentment", a meaningful name. Links the feeling of "sufficiency", especially regarding children and livelihood, to personality as a feeling of lasting peace. |
|  | *Madimabe* | "Bad luck", a meaningful name. Identifies adversities with personal fate, functioning both as self-pity and as a social warning. |
|  | *Mahlomola* | "Suffering, loneliness, bewilderment", a meaningful name. Biographies marked by profound losses brand individuals with an enduring identity of pain and loneliness. |
| Lebaka (2020) | *Malopo* | Music/dance rituals through which contact is made with Bapedi ancestral spirits. By interpreting illness and "ill omens" as an ancestral calling, transforms fear, guilt, and hope into the collective dramaturgy of healing. |
| Lotsu (2025) | *Nwomkro* | An Akan dirge/lament genre performed at funerals. Performatively produces mourning, longing, and solidarity by moving individual loss onto the stage of collective memory and emotionality. |
|  | *Adowa* | An Akan funeral/festival dance-music genre. Transforms grief, pride, and resilience through body and rhythm, turning death into the aesthetic stage of communal endurance. |
| Luka and Martin (2025) | *Kajju* | The name the Bajju people give to their own land; carries the feeling of "our place." Establishes place-based pride and belonging; links land loss to a deep sense of injustice and resistance. |
| Madlome (2022) | *Ubuntu / Botho / Vumunhu* | "Humanity, humanness" in South African Bantu languages; defines "being a good person" through compassion, solidarity, and mutual care. Functions as the overarching ethical-emotional principle normatively framing emotions (pride, shame, anger) in favor of the community. |
| Madlome (2022) | *Makarushi* | A derogatory ethnic nickname externally attributed to the Vatsonga implies devaluation and animalization. Reproduces colonial hierarchy and stigmatization at the emotional level by generating shame, anger, and counter-pride. |
| Memela et al. (2025) | *Ubuhlanti* | An integral part of the male circumcision ritual known as 'ulwaluko' among amaXhosa, performed during the transition of young men from adolescence to adulthood and their acceptance into manhood. It depicts a place such as a house or barn. This visit is associated with manhood, courage, and fearlessness. |
|  | *Ixhanti / iinxhanti* | A ritual space/fences around Ubuhlanti, the site of votive, purification, and protection rituals. Concentrates respect for ancestors, anxiety, and piety, placing them in place and transforming emotional tensions into ritual expression. |
|  | *Ulwaluko* | The Xhosa male initiation/circumcision process; involves pain, solitude, and risk alongside honor, courage, and the pride of being accepted. A rite of passage that sets the emotional threshold of "becoming a real man"; regulates fear and shame according to community norms. |
| Michael and Mensah (2025) | *Chínènyéńwā* | "God gives a child" a meaningful name; positions birth as a gift and grace from God. Establishes feelings of gratitude and humility alongside joy; organizes the identity of child and family around thankfulness. |
|  | *Chíkàódìnáká* | The expression "everything is in God's hands"; language of submission linking fate and daily events to divine control. Offers a cognitive-emotional framework that converts anxiety and helplessness into receptive hope. |
|  | *Ókwúdìríchúkwú* | "I leave revenge to God"; expresses delegating punishment to God in the face of injustice. Functions as an emotional "valve" that limits personal revenge and violence by delegating anger to divine justice. |
|  | *Chínwèíkpé* | "Revenge belongs to God"; emphasis on divine justice that prohibits personal retaliation. Legitimizes feelings of patience and waiting by supporting a sense of protection and anger control in situations of victimization. |
|  | *Nwósū / Òsúàlà / Òsújī / Òsúágwù* | Meaningful Òsú caste names meaning "outcast/slave of the earth, the yam, the god"; signal permanent exclusion. Naming practices that institutionalize feelings of shame, fear, and worthlessness throughout the life cycle. |
| Mire (2015) | *Wagar* | A sacred wooden figure/object associated with fertility in the Somali context; carries hope for abundance and protection. Directs infertility anxiety toward a concrete object, transforming fear and helplessness into ritual hope and reassurance. |
|  | *Waaq* | The sky God in Somali/Oromo tradition; the source of rain, fertility, and fate. Regulates joy and disappointment within a theological-emotional framework by linking climate and livelihood fluctuations to divine will. |
|  | *Nagi* | A term expressing peace, tranquility, and social harmony; the ideal state of the community. Constrains feelings of conflict, gossip, and jealousy through the fear of "breaking nagi." |
| Mire (2016) | *Baanashada Dumarka* | A fertility/healing ritual specific to women; involves incense, plants, touch, and prayer. Transforms shame and sorrow regarding infertility and marital crisis into feelings of hope and strength within women's solidarity and care. |
| Mire (2016) | *Tiire* | A plant in Baanashada attributed with fertility and protective power. Converts anxiety into a feeling of concrete protection by providing a bodily sense of a "shield"; the emotional symbol of healing. |
|  | *Zar* | A spirit possession and trance complex; a practice in which distress is explained through spirits taking over the body. Provides relief by allowing suppressed anger, sorrow, and trauma to be expressed and negotiated in front of the community. |
| Mthembu (2020) | *Ilobolo / ukulobola* | Zulu bridewealth; a marker not merely of economic value but of respect, responsibility, and inter-family bonds. A relational practice that emotionally constructs masculine pride and the duty of care. |
|  | *Umshado* | Wedding ceremony; celebrated with dance, song, and gifts. Performatively intensifies feelings of joy, prestige, and unity for families and the community. |
|  | *Umabo* | A gift/offering ritual performed on the bride's return to her family. Rebalances debt, embarrassment, and honor by softening resentment and hierarchical tensions through performances of generosity and gratitude. |
| Mukosi et al. (2025) | *Ṅwana u rinwa u ya nga zwine vhabebi kana mubebi a vha a tshi khou ḓipfhisa zwone* | Children are named according to what the parents are feeling. This reveals that emotions are important for parents when naming a newborn baby and should therefore not be disregarded in the Vhavenda community. |
|  | *Athifhelimbilu* | "I am not worried", a meaningful name. Personal names among the Tshivenda represent intra-family conflict, luck, and reincarnation. |
|  | *Mashudu* | "Luck", personal names among the Tshivenda represent intra-family conflict, luck, and reincarnation. |
| Ngcobo (2020) | *amadlozi / badimo* | Zulu/Sotho ancestors; the central source of feelings of reverence, fear, gratitude, and guilt as invisible family members and protective spirits. |
|  | *Umsamu* | A sacred corner/hearth in the home dedicated to ancestors; simultaneously intensifies feelings of sacred awe and domestic security and intimacy, drawing an emotional analogy with the space of the Communion altar. |
|  | *Umphako* | "Provisions for the journey" placed with the deceased at burial; makes feelings of grief at parting, love, and hope tangible by constructing an image of a nourishing bond with the afterlife. |
|  | *İsiphandla* | A bracelet made from the hide of the sacrificial animal; a continuous bodily sign of votive offering and devotion to the ancestors, carrying feelings of sacred belonging and pride. |
|  | *İzithakazelo* | Clan praise names; elevates collective feelings of pride and belonging by invoking ancestors and lineage history in rhythmic speech. Carries the teaching that one who knows their past knows themselves. |
| Ngubane and De Gama (2024) | *Ufufunyana* | Distress conceptualized as spiritual/ancestral attack or possession in the Zulu context; situates feelings of terror, helplessness, and shame within a social/spiritual explanation, generating shared meaning and hope for healing. |
|  | *Amadlozi* | Ancestors; organizes feelings of fear, guilt, and hope as both the cause of ufufunyana experiences and their potential source of healing. |
|  | *uMvelinqangi* | "The first/primordial one"; the Supreme Being, the source of cosmic order; structures feelings of awe, submission, and trust regarding expectations of fate, justice, and healing. |
| Ohaja and Anyim (2021) | *omugwo* | A postpartum ritual in which the mother is cared for and protected by female relatives for an extended period; acknowledges maternal status, regulating feelings of vulnerability and joy through collective care, compassion, and solidarity. |
| Oyelade and Olumiyiwa (2023) | *Aladura* | "Those who pray"; identifies Yoruba healing and spiritual warfare churches, transforming individual fear and helplessness into the ecstasy of communal prayer and feelings of trust in divine power. |
|  | *Awon Afadura j’agun* | "Prayer warriors"; a community of believers devoted to spiritual warfare; produces feelings of militant faith and collective empowerment by organizing threat, anger, and courage within the frame of "holy war." |
|  | *Tibitire ni a da ile aye* | "The reality of life lies in the unity of good and evil"; expresses that the world contains both good and evil within itself. |
|  | *Eyinkule l’ota wa, inu ile laseni ngbe* | "The enemy is outside the door, but the real enemy is inside the house"; feeds suspicion, fear of betrayal, and resentment toward those closest, emotionally reinforcing the image of the "hidden enemy." |
|  | *Ara ile eni ni ota eni* | "A person's real enemy is their own household"; frames intra-family jealousy and conflicts, linking distrust and anger to the discourse of spiritual warfare. |
|  | *Ogun l’aye, ile aye ogun ni* | "Life is a battle, the world is a battleground"; normalizes a constant sense of alertness and being under attack, emotionally recoding all hardships as part of a cosmic battle. |
|  | *Warfare prayer* | Yoruba hymns invoking God's warrior power; elevates collective fervor through bodily movement and loud voices, transforming fear and anger into feelings of "legitimate defense" and victory. |
| Petrollino (2025) | *tɔ́ɔla* | A herding imitation game played by Hamar children with stones and fruits; by combining pride of responsibility, mild anxiety, and the joy of play, it embodies an emotional bond with cattle and a sense of pastoral responsibility from an early age. |
|  | *garánti* | A simple herding game played with Solanum incanum fruits; nurtures feelings of ownership, everyday security, and joy, establishing a sense of bond with the herd and a feeling of competence in young children. |
|  | *yígi* | The general category of "play"; gamifies the emotional burden of economic activities for children, producing a feeling of resilience by associating arduous pastoral life with pleasure and pride. |
|  | *deelíndar maatá* | The homecoming phase; allows children to experience feelings of relaxation and belonging after an intense day, through the metaphor of a safe return together with the herd. |
| Rasmussen (2022) | *ark eghaghel / eghaghel* | In Tamajaq, a general category meaning "bad work, bad deed," translated into French as "sorcellerie" (sorcery); covers covert acts of power such as harming, causing illness, and killing; shapes regimes of emotional insecurity by linking fear, suspicion, jealousy, and paranoia to social crises and inequalities. |
|  | *Mahiba* | The ark echaghel type, meaning "love magic," aims to make the target involuntarily fall in love/become bound; it codes love as a political process involving ritual and power relations rather than free will, framing romantic desire and passion under the shadow of manipulation and fear. |
|  | *togerchet / tehot* | "Evil eye, malevolent eye"; the power of envy and jealousy to cause harm through the gaze; conceptualizes jealousy as a cosmic force with concrete damage potential in contexts of economic and status tension, always coding success and prosperity alongside potential attack and fear. |
|  | *alheri* | "Goodness, grace, charity, blessing"; enables the conceptualization of good fortune and happiness as spiritual grace; frames happiness as an emotional indicator of alignment with cosmic/moral order rather than individual achievement. |
| Roba (2022) | *horooroo afataa* | A ritual in Guji Oromo society in which people lay down their sticks and pray collectively in the face of major threats such as epidemics, famine, and war; transforms fear and collective anxiety into prayer and solidarity directed toward Waaqa, generating feelings of protection, hope, and submission in the community. |
|  | *Hulluuqaa* | A purification ritual in which people pass one by one under bunches of vines/plants fixed to the ground, shedding misfortune and "contamination" from themselves; converts feelings of contagious ill fortune and disgust into feelings of relief and new beginnings through the bodily act of passage. |
|  | *bita gatiisa* | A protective ritual performed with the intention of "turning" the direction of disasters "elsewhere"; reduces feelings of helplessness in the face of fate, reframing the perception of threat through a sense of collective action, prayer, and ritual control. |
| Singh and Bhagwan (2020) | *Inyanga* | A Zulu traditional healer/herbalist; the figure consulted in cases of illness, bad luck, and spiritual distress; provides feelings of hope and trust by giving meaning to individual helplessness and anxiety within a framework of invisible causes and herbal/spiritual treatment. |
|  | *İsangoma* | A Zulu medium/healer who communicates with ancestors; links bad luck, anxiety, fear, and guilt to ancestral and magical relations and transforms them through ritual negotiation, regulating the community's spiritual-emotional crises. |
| Siweya et al. (2018) | *Xivuri* | "Uncircumcised male" in Xitsonga; a derogatory label applied to young men who have not undergone traditional circumcision; draws the boundary between "a real man" and "an incomplete man" through feelings of shame, exclusion, and humiliation, tying masculine ontology to ritual. |
| Stambach and Pesambili (2025) | *Kutheni sele besomba amangcwaba ethu? (isiXhosa)* | The expression "Why are they already digging our graves?"; equates COVID-19 vaccines and state policies with death threats and betrayal, mobilizing fear, distrust, and political anger in the community around an intense grave metaphor. |
| Turner (2020) | *Dīwān* | A ritual involving music, trance, and communication with spirits; a performative emotional space that transforms trauma, grief, and fear into collective healing. |
|  | *Ihsās* | Feeling and sensation; conceptualizes emotion as a vibrational process circulating in the triangle of body–space–spirit. |
|  | *Jnūn / jinn* | Spiritual entities; relational actors and emotional influencers of emotions such as anger, grief, and fear. |
|  | *Jedba* | Ecstasy/trance; the threshold state in which pain and grief are transformed into intense emotional release and healing through ritual music. |
|  | *Ghabina* | The heavy emotional burden created by historical violence and deprivation; collective pain transformed through ritual. |
| van Beek (2025) | *Va (rain)* | "Rain" in Kapsiki; the focal point of feelings of fertility–life–power, and simultaneously a source of fragile hope and sacred fear. |
|  | *Rain hunt (ritüel pratik)* | A performative ritual in which young men walk, make music, and perform bodily actions to call forth rain; a mechanism for regulating collective anxiety and hope. |
